# The impact of preimplantation genetic testing on the quality of life of people undergoing assisted reproduction treatment

**DOI:** 10.1038/s41598-026-45746-0

**Published:** 2026-03-23

**Authors:** Maria Alciene Saraiva de Souza, Erik Montagna, Caio Parente Barbosa, Bianca Bianco, Victor Zaia

**Affiliations:** 1 Postgraduate Program in Health Sciences, Centro Universitário FMABC, Santo André, Brazil; 2 Ideia Fértil Institute of Reproductive Health, Santo André, Brazil

**Keywords:** Assisted reproductive technique, Affective aspects, Preimplantation genetic testing, Quality of life, Mental health, Psychology, Health care

## Abstract

Approximately 20% of infertility cases have a genetic origin. Preimplantation genetic testing (PGT) is an early prenatal diagnostic tool to prevent genetic disorders through embryo selection. Given its complexity, PGT requires a multidisciplinary approach, including psychosocial support, as infertility impacts mental health and overall well-being. Patients undergoing PGT often face prior miscarriages or IVF failures, elevating negative emotions and reducing quality of life. To compare affect levels, need for parenthood, and quality of life in IVF patients with and without PGT indications. This observational cross-sectional study included 375 patients divided into ‘PGT’ (n = 73) and ‘non-PGT’ (*n* = 302) groups. Participants completed the Positive and Negative Affect Schedule (PANAS), Need for Parenthood scale, and Fertility Quality of Life tool (FERTIQOL). Group comparisons were conducted. The groups did not differ in terms of gender, type and cause of infertility, and psychotropic medication use. The PGT group reported higher negative emotions, lower quality of life compared to the non-PGT group. Both groups showed high levels of need for parenthood. Patients indicated for PGT require greater support and monitoring, experiencing a significant emotional impact from the outset of treatment.

## Introduction

The World Health Organization (WHO) estimates that 15% of couples of reproductive age experience infertility in the world^1^. From a psychosocial perspective, infertility disrupts individual development by imposing a loss of control over one’s existence, leading to various emotional responses and altered perceptions of identity^2^. An inverse relationship exists between mental health and infertility, resulting in reduced overall well-being and feelings of guilt^3–7^.

Assisted reproductive techniques such as in vitro fertilization (IVF) are available to individuals trying to conceive^8,9^. The chances of achieving pregnancy with each treatment range from 30 to 40%, depending on the type of infertility, the age of the patient, and the type of assisted reproductive technology (ART) used^10^. Throughout the treatment process, healthcare teams must be attentive, as individuals seeking to conceive who do not achieve the desired success experience unhappiness, mental and existential frustration, low social support, and worsening emotional distress with each unsuccessful attempt^11–13^. Consequently, biological infertility can lead to social sterility, and, considering that social support is related to better mental health, individuals may experience greater psychosocial impairment^13,14^.

It is estimated that 20% of infertility cases are of genetic origin and include chromosomal abnormalities and gene variants^15^. Compared to diagnoses of other origins, the genetic diagnosis of infertility has specific repercussions, such as greater difficulty in discussing one’s reproductive history and increased concerns about how possible genetic alterations could influence offspring^16^.

In cases of genetic diagnosis, couples undergoing infertility treatment may need to undergo Preimplantation Genetic Testing (PGT), which involves prenatal diagnosis to prevent genetic diseases/alterations through the selection of healthy embryos. PGT can be classified into PGT-A (aneuploidies), PGT-M (monogenic diseases or specific conditions), and PGT-SR (structural chromosomal rearrangements). PGT may also be recommended in cases of advanced maternal age and recurrent miscarriage^17–19^.

However, understanding the different techniques and objectives of PGTs may not be straightforward for the patient, or may not occur at all, particularly because of a lack of familiarity with technical terminology. This results in a limited understanding that is primarily focused on undergoing a genetic test and its general consequences^20–23^.

In ART, when undergoing PGT, an individual is informed of the evaluation results for each embryo. Embryos with normal chromosomal content and without genetic variants are eligible for transfer to the uterus or cryopreservation, whereas the affected and unviable embryos may be discarded. Therefore, couples recommended for PGT, besides undergoing an additional step in their treatment, may not be able to proceed to embryo transfer if no embryo is normal, thus interrupting treatment and the possibility of attempting conception^24^.

Given the complexity of the information and consequences involved, interdisciplinary genetic counseling is essential. This process involves informing individuals about the occurrence or risk of recurrence of a genetic condition, which may derive from embryo formation, or even arise from family and helping them understand the medical, psychological, and familial implications of the condition. It is crucial to provide relevant information that enables individuals to make autonomous decisions, allowing them to assess the consequences of their chosen actions^16,25,26^.

In an interdisciplinary team, the presence of a psychologist is essential with interventions aimed at providing support and emotional balance for the patient, especially because genetic screening may trigger anxiety, guilt, or stress during waiting periods or upon receiving the results^27^. Considering individualities, culture, and understanding of parenthood has a positive influence on outcomes and treatment adherence, reducing emotional distress and negative affect^14,28–30^.

Among the factors of emotional well-being that should be observed by an interdisciplinary team are negative affect (a measure of discomfort and disturbance in life), positive affect (a measure of enthusiasm for life)^31,32^, and quality of life (QoL), which refers to an individual’s perception of various aspects that constitute their QoL and tends to be negatively affected by infertility^33^.

Therefore, this study compared the affective aspects, need for parenthood, and QoL of individuals undergoing IVF treatment with or without an indication for PGT.

## Methods

This cross-sectional observational study included couples undergoing ART at the Ideia Fértil Institute of Reproductive Health, Center for Human Reproduction and Genetics at the Centro Universitário FMABC, Santo André, Brazil. The conception, analysis, interpretation of data, writing, and revisions followed the STROBE protocol (STrengthening the Reporting of OBservational studies in Epidemiology)^34^. The data were collected between March 1st, 2019, and April 30th, 2021.

### Participants

A total of 400 patients were invited to voluntarily participate in the study. Recruitment was conducted in person by the first and last author, directly at the clinic during the patients’ initial consultation with the healthcare team. Among them, 375 accepted and completed the questionnaire, whereas 6.25% declined participation due to lack of interest. The participants were initially divided into two groups: those who had an indication for PGT (*n* = 73) and those who did not have such indication, “non-PGT” (*n* = 302). Patients diagnosed with cancer, undergoing IVF treatment for fertility preservation, single-parent families, or homoaffective relationships were excluded from the study because of motivations for treatment other than the infertility diagnosis. As inclusion criteria, we considered: age equal to or greater than 18 years old; having started first IVF treatment; having no prior diagnosis of mental disorders; and being able to complete the study questionnaires independently.

The indication for PGT was determined based on the clinical evaluation conducted by the medical team. The main criteria included advanced maternal age, history of recurrent miscarriages, the presence of known genetic conditions or chromosomal abnormalities (such as translocations or inversions), and the diagnosis of monogenic diseases. These criteria are consistent with the recommendations of major international guidelines and the relevant scientific literature^17–19,26^.

### Instruments

- ***Positive and Negative Affect Schedule*** (PANAS), Brazilian Portuguese version^35,36^: 20 items representing specific feelings or emotional states. Each item was scored on a Likert scale ranging from 1 to 5, where 1 represented “not at all” and 5 represented “very much,” according to how the participant felt about infertility and IVF treatment. Ten items relate to negative affect, and ten relate to positive affect. The scores range from 10 to 50 points for each type of affect.
- ***Fertility Quality of Life*** (FERTIQOL), Brazilian Portuguese version^37^: This self-administered scale encompasses four major domains of human life: emotional, mind-body, relational, and social. It consists of 26 questions distributed across these domains. Each question was answered on a Likert scale from 1 to 5, with options ranging from extreme satisfaction to extreme dissatisfaction. The scores for each domain range from 0 to 100 points.
- **Need for Parenthood**: based on the ‘Fertility Problem Inventory’, Brazilian Portuguese version^38,39^, which measures emotional experiences related to infertility issues. It comprises seven questions with qualitative closed-ended responses developed to inquire about the intensity of desire for parenthood. For each question, there are seven possible responses, ranging from A = “completely false for me” to G = “completely true for me.” The results are tallied on a point scale ranging from 7 to 49, with higher scores indicating a greater desire for parenthood.

### Statistical analysis

The obtained data were transcribed into Excel and independently verified by two authors (VZ and EM). Analyses were conducted using the Python and R programming language. Descriptive statistics (means, standard deviations, medians, interquartile ranges, frequencies, and percentages) were calculated. The normality of continuous variables was assessed using the Kolmogorov-Smirnov test, which indicated a non-normal distribution. Group comparisons (PGT and non-PGT and/or men and women) were conducted using chi-square and Mann-Whitney U tests, since it is a nonparametric test, it relies on rank comparisons, thereby mitigating distortions arising from numerical differences between groups^40–42^. The interpretation of results was based on the significance level established at *p* ≤ 0.05. To compare patients undergoing PGT and those without PGT indication across the psychosocial and quality-of-life outcomes, we applied non-parametric analyses of covariance based on ranked data (rank-ANCOVA)^43^, Conover & Iman, 1981). This approach enabled us to evaluate group differences while statistically adjusting for age and history of miscarriage, thereby reducing potential confounding effects attributable to these variables. For each outcome variable (PANAS Positive, PANAS Negative, Fertiqol Emotional, Fertiqol Mind–Body, Fertiqol Relational, Fertiqol Social, and Need for Parenthood), ranked values were computed and entered into general linear models including study group (PGT vs. non-PGT) as a fixed factor and ranked age as covariate. Effect sizes were estimated using partial eta squared (η²ₚ), interpreted as small (≥ 0.01), medium (≥ 0.06), and large (≥ 0.14). Estimated marginal means of ranked outcomes adjusted for age were calculated for each group to interpret the direction of effects. To control for multiple testing across the seven outcomes, p-values were corrected using the Benjamini–Hochberg false discovery rate (FDR) procedure.

### Ethical aspects

This study was approved by the Research Ethics Committee of *Centro Universitário FMABC/Faculdade de Medicina do ABC*, with national validity (protocol number: 88192218.3.0000.0082/2.675.077). Additionally, the present study adhered to the principles of the Declaration of Helsinki. All participants were volunteers. They were all of legal age and were invited in person by the first and last authors. The study objectives were explained to them, and upon consenting to participate, they signed a consent form, which was archived by the last author. Refusal to participate in the study did not result in any detriment to their treatment.

## Results

A total of 375 patients who underwent IVF participated in the study. They were divided into two groups, PGT (302 participants) and non-PGT (73 participants). The only variables showing differences between the groups were history of miscarriage and age, both more frequent in the PGT group, whereas no significant differences were observed for the other characteristics (Table 1).

**Table 1:** Characteristics of study patients and comparison between groups.

Variables		non-PGT *n* (%)	PGT *n* (%)	*p*-value*
N		302 (80.5)	73 (19.5)	
Gender	Female	150 (49.7)	43 (58.9)	0.157
	Male	152 (50.3)	30 (41.1)	
Income range				
Up to US$572,00		6 (0.0)	1 (1.37)	0.937
573–1144		139 (46.0)	28 (38.36)	
1145–1716		151 (50.0)	32 (43.84)	
More than 1716		6 (2.0)	2 (2.74)	
Education level				
Middle School		17 (5.6)	7 (9.9)	0.205
High School		95 (31.5)	16 (22.5)	
Technical High School		16 (5.3)	38 (50.7)	
Higher education		141 (46.7)	6 (8.5)	
Graduate Studies		33 (10.9)	6 (8.5)	
Psychotherapy		22 (7.3)	13 (17.8)	0.059
Psychotropic medication		12 (4.0)	7 (9.6)	0.096
History of miscarriage		28 (9.3)	18 (24.7)	0.001
Duration of infertility (years)		3 (2.0)	3 (2.0)	0.507
Previous pregnancy	No	229 (75.8)	48 (65.8)	0.107
	Yes	73 (24.2)	25 (34.2)	
Belief in Success Probability	Up to 40%	54 (17.9)	15 (20.5)	0.598
	More than 40%	248 (82.1)	58 (79.5)	
Mood Changes Due to Treatment	Unchanged	164 (54.3)	41 (56.2)	0.187
	Worsened	127 (42.1)	26 (35.6)	
	Improved	11 (3.6)	6 (8.2)	
		Median (IQR)		p-value**
Age (years)^***^		34.0 (6.0)	36.0 (7.0)	0.001

PGT: Preimplantation Genetic Test. *Chi-square test. ^**^Mann-Whitney U test.

The assessment instruments for affectivity and quality of life are summarized in Table 2. Overall, the non-PGT group obtained higher scores for positive affect, quality of life across all domains, and Need for Parenthood compared to the PGT group. The rank-ANCOVA analyses, with age included as a covariate, showed that age significantly influenced only Need for Parenthood (*p* = 0.008) and the FertiQoL-Relational domain (*p* = 0.029). For Need for Parenthood, adjustment for age eliminated the previously observed group difference (F = 3.25, *p* = 0.072). In contrast, for the FertiQoL-Relational domain, the difference between PGT and non-PGT patients remained significant after adjustment (F = 11.07, *p* < 0.001), with higher scores in the non-PGT group. For all other variables (PANAS Positive, PANAS Negative, FertiQoL-Emotional, FertiQoL-Mind–Body, and FertiQoL-Social), group differences persisted independently of age, consistently indicating poorer outcomes in the PGT group. Descriptive comparisons further revealed large to very large effect sizes. The non-PGT patients reported substantially higher levels of positive affect (d = 1.46, 95% CI [1.18, 1.73]) and lower negative affect (d = − 1.19, 95% CI [–1.45, − 0.92]) than the PGT group. Similarly, non-PGT participants showed higher scores in FertiQoL-Emotional (d = 1.05), Mind–Body (d = 1.21), and Social domains (d = 1.92), with effect sizes ranging from large to very large. A medium effect size was observed for the Relational domain (d = 0.58), while the difference for Need for Parenthood was small to moderate (d = 0.37). These findings are consistent with the rank-ANCOVA analyses adjusted for age, which confirmed significant group differences in all domains except for Need for Parenthood, where the effect of age accounted for the initial discrepancy. History of miscarriage did not show any association with the tested outcomes (all p-FDR ≥ 0.59), confirming that the observed group differences were independent of this variable.

**Table 2:** Comparison of affects and quality of life domains between the studied groups.

Instrument	Domain	non-PGT	PGT	*p*-valueᵃ	F (rank-ANCOVA)	p_FDR (age-adjusted)	η²ₚ	Cohen’s d (95% CI)	Direction
PANAS*	Positive	33.0 (10.0)	22.0 (6.0)	≤ 0.001	128.44	< 0.001	0.26	1.46 (1.18–1.73)	PGT < non-PGT
	Negative	14.0 (8.0)	22.0 (6.0)	≤ 0.001	94.30	< 0.001	0.20	-1.19 (-1.45- -0.92)	PGT > non-PGT
FertiQoL**	Emotional	79.2 (29.2)	58.3 (35.4)	≤ 0.001	50.90	< 0.001	0.12	1.05 (0.78–1.32)	PGT < non-PGT
	Mind-Body	83.3 (25.0)	58.3 (29.2)	≤ 0.001	66.95	< 0.001	0.15	1.21 (0.94–1.48)	PGT < non-PGT
	Relational	83.3 (20.8)	75.0 (37.5)	≤ 0.001	11.07	= 0.001	0.03	0.58 (0.32–0.84)	PGT < non-PGT
	Social	79.2 (25.0)	45.8 (22.9)	≤ 0.001	135.98	< 0.001	0.27	1.92 (1.62–2.21)	PGT < non-PGT
Need for Parenthood		41.0 (9.0)	39.0 (12.0)	0.017	3.25	0.072	0.01	0.37 (0.12–0.63)	ns

Notes. Values are expressed as median (interquartile range). Score range: 10 to 50 points for PANAS; 0 to 100 points for FERTIQOL and 7 to 49 points for Need for Parenthood. Group comparisons were first conducted using Mann–Whitney U tests (p-valueᵃ). To adjust for age, non-parametric analyses of covariance (rank-ANCOVA) were performed, reporting F statistics, false discovery rate (FDR)-adjusted p-values, and partial eta squared (η²ₚ) as effect size. Estimated marginal means of ranked outcomes adjusted for age indicated the direction of differences (PGT vs. non-PGT). Cohen’s d was calculated as a standardized measure of effect size for the unadjusted group comparisons, with 95% confidence intervals. * Positive and Negative Affect Schedule (PANAS). ** Fertility Quality of Life (FertiQoL) questionnaire.

**Fig. 1:**
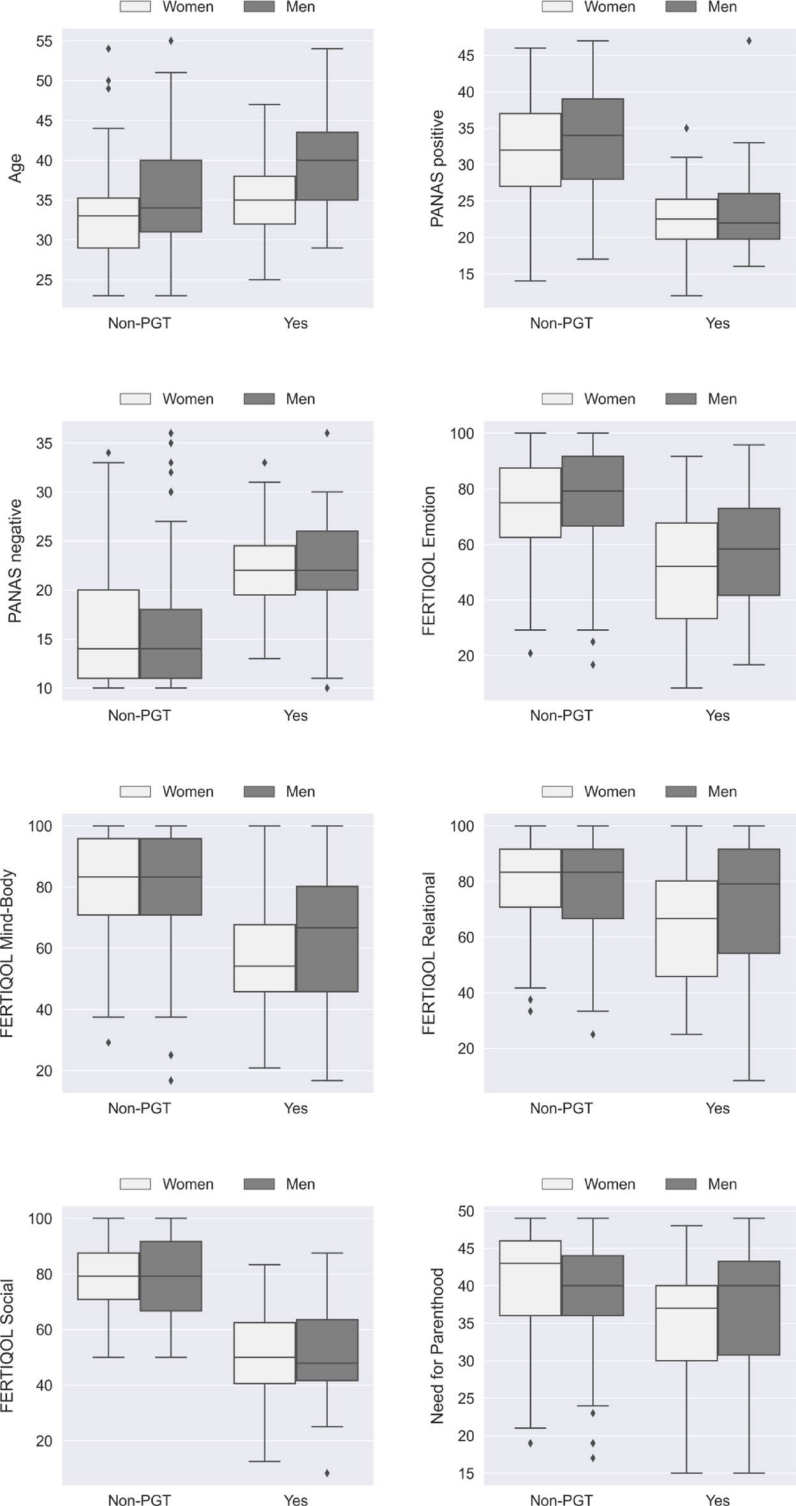
Boxplots comparing the PGT and non-PGT groups for the variables age, affect, quality of life, and need for parenthood. Notes: Boxplots of observed scores for age, PANAS (positive and negative affect), domains of the Fertility Quality of Life – FERTIQOL (Emotion, Mind-Body, Relational, Social), and Need for Parenthood. The plots display the distribution of the variables across non-PGT and PGT groups, stratified by gender (women = white boxes; men = gray boxes). The horizontal line within each box represents the median; the lower and upper limits of the box correspond to the first and third quartiles; whiskers extend up to 1.5 times the interquartile range. Diamonds indicate outlier values.

Figure 1 shows the boxplots comparing the PGT and non-PGT groups, considering the subgroups of women and men. The non-PGT group had higher scores on positive affect, quality of life, and the need for parenthood, whereas the PGT group had higher scores on age and negative affect. Considering the differences between the male and female subgroups, only the differences in the non-PGT group regarding age (with men being older, *p* = 0.005) and need for parenthood (with higher scores in women, *p* = 0.022) were significant.

## Discussion

This study aimed to verify and compare the affect and quality of life in human reproduction patients who did or did not have an indication for PGT. Thus, no group differences were observed for characteristics that traditionally influence psychometric measures and mental health, such as gender, type, duration and cause of infertility, socio-economic status, use of psychotropic drugs, and undergoing psychotherapy^13,14,29,33^.

The incidence of previous miscarriage and age differed between groups, both with a higher incidence in those with an indication for PGT, an expected finding, considering these are reasons to undergo PGT^18,44^. To further examine their potential role, age and history of miscarriage, which differed between groups, were examined as potential covariates in relation to the variables of interest (affect, quality of life, and need for parenthood). Analyses indicated that these differences did not affect the measures, except for need for parenthood, for which the initial group difference disappeared after adjustment for age. Moreover, the chosen psychometric instruments allow us to access measurements at a specific moment in the participant’s life, namely, at the time of infertility treatment^35,37,38^. The difference in sample size between the PGT and non-PGT groups reflects the characteristics of the overall sample of individuals with infertility, wherein only 20% of the cases presented with some genetic diagnosis^15^.

The PGT group had lower levels of quality of life, positive affect than the non-PGT group, indicating that this group presents greater psychosocial vulnerability within the treatment process, which is consistent with previous research findings^16,45^. Additionally, all participants were at the beginning of human reproductive treatment when they took part in this study, showing that emotional well-being may differ not only according to the treatment itself, but also according to the additional step of having an indication for PGT^4^. Thus, PGT appears to be negatively associated with the psychological variables of these individuals.^46^.

The success rate of human reproductive treatments is approximately 40%; nevertheless, it was found that most participants, regardless of the group, held a belief in treatments exceeding this percentage, demonstrating heightened expectations regarding the treatment’s positive outcomes. Subsequently, this can lead to increased frustration^10^. In the PGT group, this heightened belief may have stemmed from the characteristics of genetic testing. Although it has a negative impact, it also allows the identification of a normal embryo with significant potential for achieving pregnancy^47^.

However, it is important to emphasize that human reproduction treatment and PGT are not able to provide certainty about the outcome to the patient^10,47^. Therefore, high expectations, as described, regarding an outcome that may not ultimately lead to the birth of a child, could exacerbate negative impacts related to emotional well-being and adaptation to new possible treatment cycles^29^.

Another characteristic exhibited by the participants, which could serve as a parameter for emotional well-being, is mood swings due to the treatment^33^. A significant portion of the participants experienced worsened mood due to the treatment, indicating that emotionally sensitive patients may struggle with the lengthy and initially non-resolutive treatment process. These data reinforce the need for emotional counseling^6,48^.

In a preliminary analysis, lower levels of need for parenthood were observed in the PGT group compared to the non-PGT group. However, this difference did not remain after adjusting for age, indicating that the variation was primarily influenced by this variable. This phenomenon was consistent with higher age being associated with a sense of hopelessness^49^, especially within the context of human reproduction, regardless of whether assisted reproductive technologies are involved^50–52^. Once age was considered, the need for parenthood was equivalent in both groups, PGT and non-PGT. This finding is particularly relevant, as it shows that regardless of treatment pathways, both populations display high levels of desire for parenthood. While understandable in the context of infertility, such elevated levels may heighten experiences of frustration and emotional distress, especially when each stage of treatment – including additional procedures such as PGT – introduces further reproductive obstacles and potential feelings of disillusionment or hopelessness regarding the expected outcomes^16,51^.

From a clinical perspective, the differences observed in quality of life and affect can be interpreted as indicators of relevant psychological conditions within the trajectory of assisted reproduction. Lower levels of quality of life and positive affect in patients with an indication for PGT were consistent with greater emotional vulnerability, a factor frequently described in the literature as being associated with difficulties in treatment adherence, frustration management, and coping with repeated IVF cycles. Interpretations of this kind are supported by the literature^4,6,13,53,54^, which consistently shows that reduced emotional well-being and impaired quality of life in infertile patients are associated with heightened psychological distress, greater risk of treatment discontinuation, and less favorable reproductive outcomes.

Our findings are also aligned with a recent review^55^ and study^56^ on psychosocial aspects of assisted reproduction, which highlights the strong relationships between different ART pathways and emotional variables such as anxiety, depression, and stress. In this context, a multidisciplinary approach that includes genetic counseling and continuous psychosocial support is essential, as it may reduce emotional distress and improve treatment adherence^6,48^. Evidence suggests that cognitive-behavioral interventions – both at the individual level and targeting relational aspects of the couple – are effective strategies to address the psychosocial challenges faced by individuals and couples undergoing ART, including those specifically related to PGT^29,32,55^. Taken together, these considerations emphasize the clinical importance of integrating psychosocial care into ART protocols to mitigate emotional burden and ultimately enhance both psychological and reproductive outcomes.

The present study has some limitations. This was a cross-sectional study, which precluded verification of causal relationships. The PGT group did not allow for its breakdown into specific subgroups because the final sample size was insufficient to meet the statistical assumptions of the tests to be used. However, the decision not to divide between types of PGT was based on the literature in the field, which indicates low health literacy among patients regarding genetic issues^20^. This study relied on self-reported measures, subject to potential bias (e.g. social desirability and recall), and included only participants who provided informed consent, which may limit sample representativeness.

The study also presents several strengths. The similarity in group characteristics allowed for a more precise analytical identification of differences and similarities in affect, need for parenthood, and quality of life, providing greater theoretical confidence in comparisons between PGT and non-PGT groups. In addition, the clear definition of inclusion criteria – such as undergoing a first IVF treatment, absence of prior mental disorder diagnosis, and ability to complete the questionnaires independently – helped ensure methodological rigor and minimized potential confounding factors in the assessment of psychological outcomes.

## Conclusions

This study contributes to the literature on PGT by examining the psychosocial aspects of reproductive patients with and without an indication for this genetic test. It verified and compared quality of life, need for parenthood, and affective dimensions between the two groups.

The findings indicate that human reproductive patients with an indication for PGT require greater emotional support throughout their treatment, as they have lower levels of quality of life, particularly in the social domain, and experience more negative effects than their counterparts without an indication for PGT. In both groups, levels of need for parenthood remained high, highlighting a sensitive aspect of the reproductive experience.

These results underscore the importance of considering psychosocial factors when evaluating patients indicated for PGT. Individuals undergoing assisted reproduction who require PGT may benefit from multidisciplinary care and genetic counseling strategies directed toward the promotion of mental health and the development of adaptive coping, which may enhance positive affect, need for parenthood, and quality of life.

Future longitudinal studies including a broader set of variables, such as social support and cultural beliefs regarding parenthood, is warranted to provide deeper insight into the dynamics of psychosocial factors across the treatment trajectory.

## Data Availability

All data will be made available by contacting the corresponding author.
